# Salt-Regulated Accumulation of the Compatible Solutes Sucrose and Glucosylglycerol in Cyanobacteria and Its Biotechnological Potential

**DOI:** 10.3389/fmicb.2019.02139

**Published:** 2019-09-13

**Authors:** Friedrich Kirsch, Stephan Klähn, Martin Hagemann

**Affiliations:** ^1^Department of Plant Physiology, Institute for Biosciences, University of Rostock, Rostock, Germany; ^2^Department of Solar Materials, Helmholtz-Centre for Environmental Research–UFZ, Leipzig, Germany

**Keywords:** cyanobacteria, compatible solute, salt stress, sucrose, glucosylglycerol, glucosylglycerol-phosphate synthase/phosphatase, sucrose phosphate synthase

## Abstract

Cyanobacteria are prokaryotes that can assimilate inorganic carbon *via* oxygenic photosynthesis, which results in the formation of organic compounds essentially from CO_2_, water, and light. Increasing concerns regarding the increase in atmospheric CO_2_ due to fossil energy usage fueled the idea of a photosynthesis-driven and CO_2_-neutral, i.e., cyanobacteria-based biotechnology. The ability of various cyanobacteria to tolerate high and/or fluctuating salinities attenuates the requirement of freshwater for their cultivation, which makes these organisms even more interesting regarding a sustainable utilization of natural resources. However, those applications require a detailed knowledge of the processes involved in salt acclimation. Here, we review the current state of our knowledge on the regulation of compatible solute accumulation in cyanobacteria. The model organism *Synechocystis* sp. PCC 6803 responds to increasing salinities mainly by the accumulation of glucosylglycerol (GG) and sucrose. After exposure toward increased salt concentrations, the accumulation of the main compatible solute GG is achieved by *de novo* synthesis. The key target of regulation is the enzyme GG-phosphate synthase (GgpS) and involves transcriptional, posttranscriptional, and biochemical mechanisms. Recently, the GG-degrading enzyme GG hydrolase A (GghA) was identified, which is particularly important for GG degradation during exposure to decreasing salinities. The inversely ion-regulated activities of GgpS and GghA could represent the main model for effectively tuning GG steady state levels according to external salinities. Similar to GG, the intracellular amount of sucrose is also salt-regulated and seems to be determined by the balance of sucrose synthesis *via* sucrose-phosphate synthase (Sps) and its degradation *via* invertase (Inv). In addition to their role as stress protectants, both compatible solutes also represent promising targets for biotechnology. Hence, the increasing knowledge on the regulation of compatible solute accumulation not only improves our understanding of the stress physiology of cyanobacteria but will also support their future biotechnological applications.

## Introduction

Cyanobacteria are the only prokaryotes performing oxygenic photosynthesis, a process which uses light energy to assimilate CO_2_ into carbohydrates and biomass, thereby releasing oxygen as byproduct from water splitting. It is commonly accepted that oxygenic photosynthesis has evolved within the group of cyanobacteria. The evolution of cyanobacteria initiated the rise of free oxygen in the ancient atmosphere, which is known as the great oxygenation event (Hohmann-Marriott and Blankenship, 2011; Lyons et al., 2014; Soo et al., 2017). Accordingly, ancient cyanobacteria had a huge impact on Earth’s biogeochemical history not only by producing the oxygen-containing atmosphere and provoking the evolution of aerobic life, but also by providing organic matter in the global food web. Moreover, an early representative of cyanobacteria served as an endosymbiont within a eukaryotic cell, subsequently establishing the chloroplast as photosynthetic organelle in eukaryotic algae and plants (Martin et al., 2015). During their long evolution, cyanobacteria developed a large variability in metabolism, morphology, and tolerance to abiotic stresses (Tandeau de Marsac and Houmard, 1993). These abilities enable cyanobacteria to colonize almost all aquatic and terrestrial habitats, as long as minimal amounts of water, light, and some trace minerals are present. To date, cyanobacteria still play an important role in global biogeochemical cycles. For example, marine picocyanobacteria of the genera *Prochlorococcus* and *Synechococcus* account for approximately 25% of primary production in the oceans (Flombaum et al., 2013). In addition to inorganic carbon, the fixation of atmospheric nitrogen by several cyanobacterial strains is a major source of reduced inorganic nitrogen compounds in marine habitats (Foster et al., 2011; Zehr and Kudela, 2011).

Due to their photosynthetic lifestyle, there is a rising interest for cyanobacteria in green biotechnology (for reviews, see Ducat et al., 2011; Savakis and Hellingwerf, 2015; Hagemann and Hess, 2018). For instance, cyanobacterial cells can be used as sustainable producers of various chemical compounds or fuel components. The interest in biofuels is mainly driven by the increasing global energy demand, which in turn forces mankind to find alternatives for the limited fossil energy reserves and the high CO_2_ emission during their utilization.

The advantage of using cyanobacteria is essentially based on three basic ideas: first, phototrophic microorganisms do not require energy to build differentiated organs, such as roots, leaves, and flowers, which promise greater efficiency in the conversion of light energy into the desired product (Mata et al., 2010). Secondly, their cultivation does not depend on agricultural land–there is no competition with the cultivation of crops for food and feed production (Ahmad et al., 2011). And thirdly, many of these organisms tolerate enhanced salinities; hence, their cultivation does not necessarily rely on limited freshwater availability (Chisti, 2013; Pade and Hagemann, 2015). In recent years, great efforts have been made to produce a wide variety of products using cyanobacteria as hosts. These include ethanol and biodiesel, fatty acids, isobutanol, isoprene, and many more (reviewed in Case and Atsumi, 2016; Singh et al., 2017). Relatively high production titers have been reported, for example, for cyanobacterial ethanol production with rates of 230 up to 270 mg L^−1^ d^−1^ (Gao et al., 2012; Dienst et al., 2014). However, albeit a variety of different approaches have been attempted; no economically competitive biotechnological production of bioenergy and chemical feedstock has yet been established with cyanobacteria. Therefore, further research is required which tackles, for instance, the design of cost-effective bioreactors. In contrast to large-scale industrial fermenters for heterotrophic bacteria, appropriate light delivery represents one of the major challenges for cyanobacterial mass cultivation. This is due to the fact that shading effects occur proportional with cell growth, which in turn limit biomass and often product formation (for a review on photobiorectors, see Posten, 2009). Nevertheless, significant progress has been made during the last two decades, for instance by using flat panel reactors in a pilot scale (e.g., Subitec GmbH, https://subitec.com/en). Furthermore, the application of new methods in the field of synthetic biology might enable more efficient delivery of carbon into the respective end product in the future (Hagemann and Hess, 2018). In addition, the stress tolerance of the used cyanobacterial strains comes to the fore considering the indispensable effects of fluctuating temperatures and changing light conditions when exposed to natural sunlight (Singh et al., 2017). This also counts for the anticipated cultivation in salty media, i.e., naturally available brackish or seawater. In this context, the tolerance of the producer strains to salt is one of the most important challenges.

### Salt Stress and the Accumulation of Compatible Solutes

Salinity is one of the major abiotic environmental factors in aquatic but also terrestrial habitats. Fluctuating salt concentrations essentially confront organisms with two problems. First, high amounts of dissolved ions decrease the osmotic potential in the surrounding medium, which has a direct influence on the water content within the cell. An increasing salinity is subsequently accompanied by an osmotic loss of water, which is conflicting the maintenance of turgor pressure as a driving force for cellular growth (Ladas and Papageorgiou, 2000). Second, especially during sudden increases of the external salinity (salt shock), which, e.g., naturally occur in river estuaries, large amounts of inorganic ions penetrate into the cell, thereby disturbing intracellular ion homeostasis. This is problematic because many cellular processes are directly or indirectly dependent on various inorganic ions such as potassium (K^+^), calcium (Ca^2+^), and magnesium (Mg^2+^). However, sodium (Na^+^) and chloride (Cl^−^) have the largest percentage of dissolved salt ions in natural saline habitats (Pawlowicz, 2013). Thus, salt stress can lead to a drastic change in the intracellular ion composition. In addition, high intracellular Na^+^ concentrations are directly toxic due to negative effects on the hydration shell and the surface charge of macromolecules (Klähn and Hagemann, 2011).

**Figure 1:**
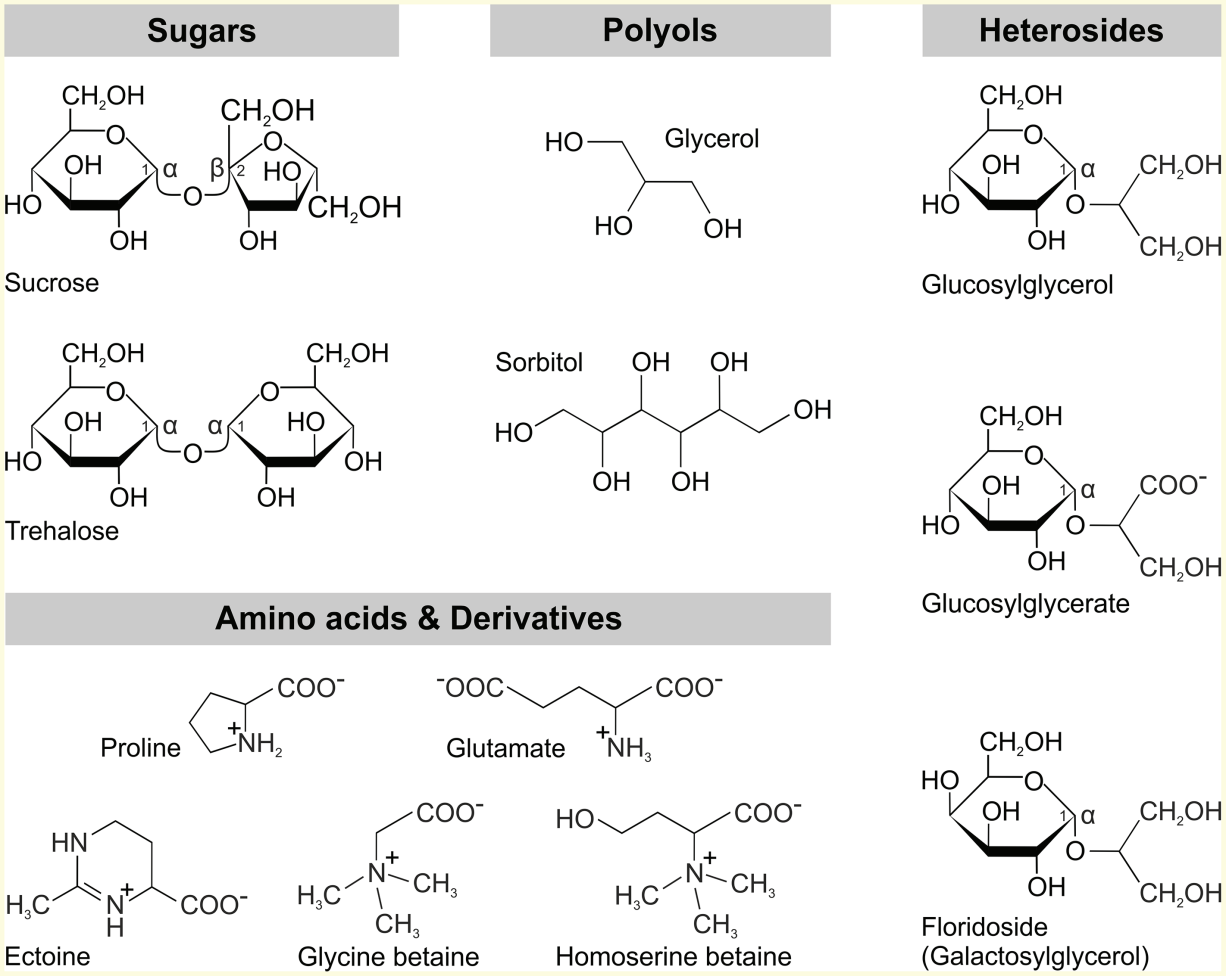
Classes of compatible solutes found in microorganisms.

To accommodate increasing salinity, most microorganisms including cyanobacteria use the salt-out strategy, which involves the active export of Na^+^ and Cl^−^ as well as the intracellular accumulation of osmoprotective organic compounds (Hagemann, 2011). The former counteracts the influx of toxic ions in the cell and serves to maintain ion homeostasis. The accumulation of osmoprotective compounds, the so-called compatible solutes, serves to balance the osmotic potential between the cell interior and exterior. Compatible solutes are low-molecular mass substances that are readily soluble in water and can be accumulated in high concentrations within the cell without interfering with metabolism and other cellular processes (Brown, 1976). By accumulating these substances, it is possible to compensate for changes in the osmotic potential and thus ensuring the uptake of water to maintain turgor and cellular growth. Structurally, compatible solutes can be assigned to different substance classes (Figure 1); these are often sugars (trehalose, sucrose), polyols (glycerol, sorbitol), heterosides (glucosylglycerol, floridoside; Hagemann and Pade, 2015) or amino acids (glutamate, proline), and amino acid derivatives (glycine betaine, ectoine). The intracellular accumulation can be achieved either by the uptake of externally available compatible solutes or by *de novo* synthesis.

Within the group of cyanobacteria, there is a rough correlation between the compatible solutes used to compensate osmotic potential and the natural habitat of the respective strain. For instance, freshwater strains with low salt tolerance usually use the disaccharides sucrose and trehalose, while in moderately salt-tolerant, e.g., marine species the heteroside glucosylglycerol (GG) is predominantly accumulated. Glycine betaine and glutamate betaine were identified as compatible solutes in halophilic species at extremely saline sites (Mackay et al., 1984; Hagemann, 2011). The preference of specific compatible solutes at different salinity levels might be correlated with the specific degree of protection for the stabilization of macromolecules such as enzymes or membranes (Borges et al., 2002; Hincha and Hagemann, 2004). Nevertheless, it should be noted that the classification into these three groups is not always reflected among cyanobacteria. Within the marine representatives, there are exceptions, for example, *Crocosphaera watsonii* exclusively accumulates trehalose (Pade et al., 2012), while most oceanic *Prochlorococcus* spp. mainly accumulate sucrose (Klähn et al., 2010a). Moreover, some *Prochlorococcus* and *Synechococcus* strains also synthesize glycine betaine as well as unusual solutes such as the negatively charged glucosylglycerate (Klähn et al., 2010a). In the important nitrogen-fixing strain *Trichodesmium erythraeum*, in turn, none of the compounds mentioned are found as compatible solutes, but this organism uses the recently identified homoserine betaine (Pade et al., 2016a).

### Cyanobacterial Salt Acclimation and Biotechnology

To increase sustainability, large scale cultivation of cyanobacteria for the biotechnological production of biofuels or bulk chemicals shall avoid the use of large quantities of limited freshwater resources and should be preferentially done in brackish or seawater (Chisti, 2013). The influence of increased salt content on the production of various substances has been scarcely investigated. A few reports are available for ethanol (Pade et al., 2017), isoprene (Chaves et al., 2015; Pade et al., 2016b), and glycogen (Aikawa et al., 2014) production in salt-stressed cyanobacteria. With the model strains *Synechocystis* sp. PCC 6803 (hereafter *Synechocystis*) and *Synechococcus* sp. PCC 7002, two moderately salt-tolerant cyanobacteria accumulating GG as main compatible solute were used in these studies. At least with respect to the mentioned compounds, an externally supplied NaCl concentration of 2% (w/v) did not significantly affect the productivity of these strains. At a NaCl concentration of 4%, which is slightly above the average salinity of seawater, the respective production rates decreased. In this regard, the high rate of GG synthesis, thereby reducing the flow of carbon into the respective product, might be crucial for the decreased product yield in these strains (Pade et al., 2017). However, the low fluxes of carbon from the basal metabolism into the desired products represent a general problem of cyanobacterial biotechnology (Angermayr et al., 2009).

In addition to the influence of salt on the production of various substances, the compatible solutes themselves are increasingly coming into the focus of a biotechnological utilization. For example, GG can be used as stabilizing agent for enzymes or antibodies, and hence enables their long-term storage in freezers (Luley-Goedl et al., 2010; Tan et al., 2015). Moreover, due to its biochemical properties, it is also commercially used in cosmetics, i.e., as a component of several skin revitalizing creams (GG is produced by the bitop AG, Germany, and designated as Glycoin^®^). Sucrose is of interest as a sweetener in food industry, as a high-energy sugar for fuel production as well as a feedstock for the cultivation of heterotrophic microorganisms (Ducat et al., 2012; Du et al., 2013; Löwe et al., 2017; Kirsch et al., 2018).

## Accumulation of Sucrose and Glucosylglycerol Among Salt-Stressed Cyanobacteria

The salt-induced accumulation of sucrose was first reported in *Synechococcus elongatus* PCC 6301 (Blumwald et al., 1983) and *Anabaena variabilis* (Erdmann, 1983). In the meantime, the widespread distribution of sucrose as a compatible solute has become evident on the basis of the numerous sequenced cyanobacterial genomes (Hagemann, 2013; Kolman et al., 2015). In fact, phylogenetic studies suggest that the synthesis of this sugar occurred very early in cyanobacterial evolution, and that sucrose was the first osmoprotective substance in ancient cyanobacteria (Blank, 2013). By acquiring the genes for the synthesis of other compatible solutes and concomitant conquest of marine and hypersaline habitats, the genes for sucrose metabolism were lost in some groups. Among cyanobacteria, sucrose remains the dominant compatible solute in freshwater habitats in addition to trehalose and also occurs in some moderately salt-tolerant strains such as *Synechocystis* as a secondary compatible solute besides GG. Presumably, the genes of sucrose metabolism were transferred to algae and plants in the endosymbiotic formation of chloroplasts (Lunn, 2002; Salerno and Curatti, 2003).

The first detection of GG in cyanobacteria was reported for extracts of *Agmenellum quadruplicatum* (former name of *Synechococcus* sp. PCC 7002; Kollman et al., 1979). Subsequently, the salt-dependent accumulation of GG and thus its function as a compatible solute could be demonstrated (Borowitzka et al., 1980). In the meantime, GG has been identified as an osmoprotective substance in more than 60 cyanobacteria (Hagemann, 2011). In many marine cyanobacteria, GG represents the primary compatible solute, but there are also numerous strains in which GG does not occur. For example, key genes of GG metabolism are absent in most strains of the ecologically important picocyanobacteria *Prochlorococcus* spp. (Scanlan et al., 2009). Only recently, the first member of this genus could be identified with an intact GG synthesis in the Red Sea (Shibl et al., 2018). Conversely, the synthesis of GG has also been demonstrated in some cyanobacteria originally isolated from freshwater habitats, including the model strain *Synechocystis* (Reed and Stewart, 1985). GG synthesis, however, is not restricted to cyanobacteria. Several heterotrophic bacteria possess a full GG synthesis pathway and accumulate GG in response to osmotic stress (Pocard et al., 1994; Roder et al., 2005).

### Enzymes of Glucosylglycerol and Sucrose Synthesis

**Figure 2:**
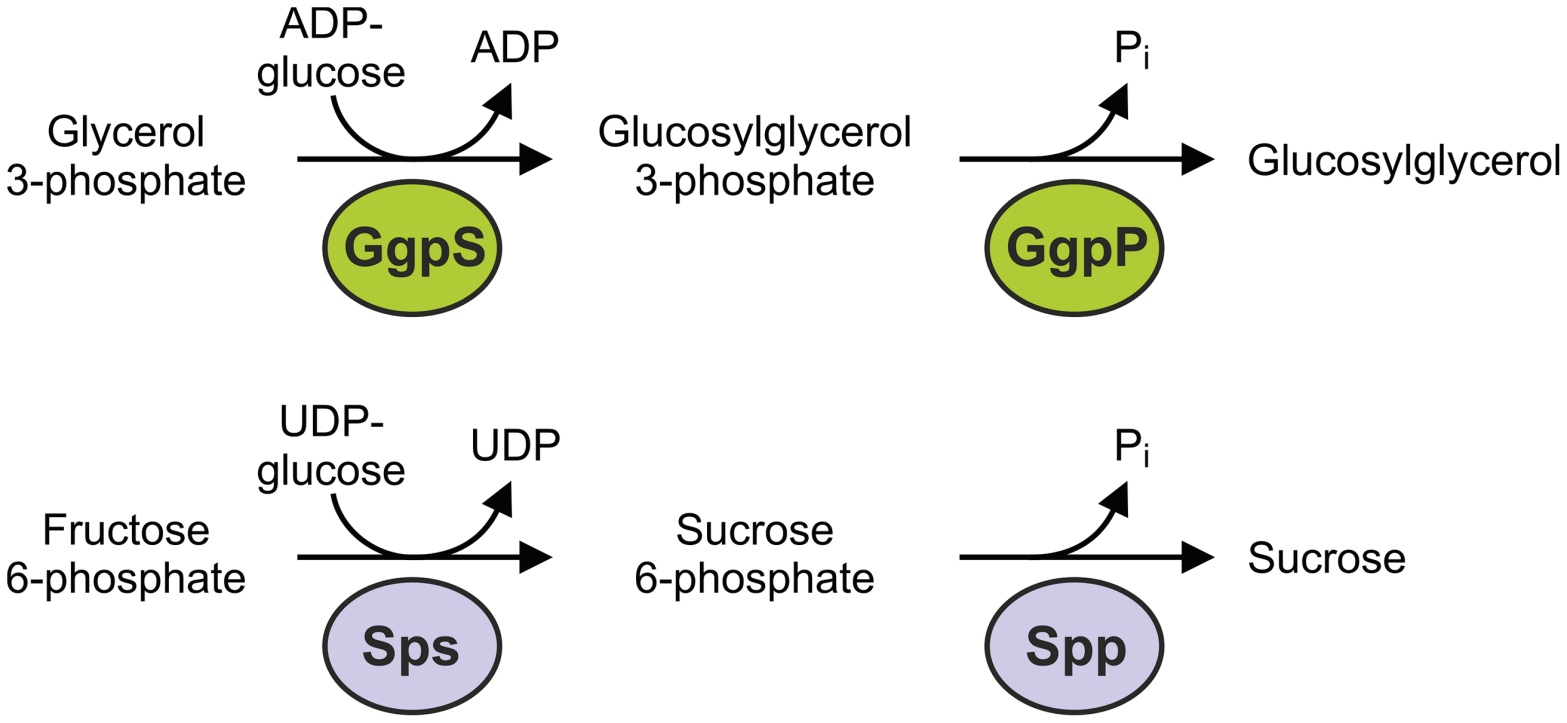
Reactions and enzymes involved in the *de novo* synthesis of glucosylglycerol (GG) and sucrose in cyanobacteria.

The synthesis of GG has mainly been investigated in *Synechocystis*. It is formed within two enzymatic steps by GG-phosphate synthase (GgpS, Marin et al., 1998) and GG-phosphate phosphatase (GgpP, Hagemann et al., 1997a). GgpS is a glucosyltransferase catalyzing the synthesis of GG 3-phosphate from ADP-glucose and glycerol 3-phosphate. This intermediate is subsequently dephosphorylated by GgpP (Figure 2; Hagemann and Erdmann, 1994). Albeit these two enzymes are synthesized at a basal level under low salt conditions, they are largely inactive and are activated after a salt shock. All GG-accumulating cyanobacteria investigated so far possess two separate genes for GgpS and GgpP, whereas some heterotrophic bacteria also harbor fusion proteins with both catalytic activities (Hagemann et al., 2008).

As in plants, the biosynthesis of sucrose in cyanobacteria is executed in a two step reaction by the enzymes sucrose-phosphate synthase (Sps, Curatti et al., 1998; Hagemann and Marin, 1999) and sucrose-phosphate phosphatase (Spp, Lunn, 2002). The Sps catalyzes the reaction of UDP-glucose and fructose 6-phosphate to sucrose 6-phosphate. Spp causes dephosphorylation and sucrose is released (Figure 2). Thus, the biosynthetic pathways of sucrose and GG are basically similar because they are formed in two enzymatic steps starting from an activated nucleotide sugar *via* a phosphorylated intermediate, which is synthesized by a glucosyltransferase. This also applies to the compatible solutes trehalose and glucosylglycerate (Klähn and Hagemann, 2011). In cyanobacteria, there are two structurally different forms of Sps. The proteins of *Nostoc* (*Anabaena*) sp. PCC 7119 and some other strains only require the glucosyltransferase domain for catalytic activity (Porchia and Salerno, 1996; Salerno and Curatti, 2003). More common are Sps proteins in which the glucosyltransferase domain is fused to an inactive phosphohydrolase domain, while the second reaction is catalyzed by a separate Spp protein. The first Sps variant appears to be exclusive in a few filamentous cyanobacteria, while the latter is present in most cyanobacteria, including *Synechocystis*, as well as algae and plants (Blank, 2013). The fusion of the glucosyltransferase and phosphohydrolase domains raises the question of whether bifunctional Sps proteins, which can catalyze the two steps in the synthesis of sucrose, can also be found in some organisms. In fact, the Sps of *Synechococcus elongatus* PCC 7942 also appears to have phosphohydrolase activity *in vitro* (Martinez-Noel et al., 2013). It is unclear to what extent the synthesis of sucrose in this strain is performed *via* this single protein, or is completed by the occurring separate Spp *in vivo*. An alternative route for the synthesis of sucrose can be catalyzed by the enzyme sucrose synthase (SuSy) through the reversible linkage of NDP-glucose and fructose with NDP cleavage (Porchia et al., 1999). In cyanobacteria, SuSy is important in heterocystous, nitrogen-fixing strains, in which it catalyzes the reverse reaction – i.e., the cleavage of sucrose to produce carbon skeletons (Curatti et al., 2002; Kolman et al., 2015).

### Salt-Induced Accumulation of Glucosylglycerol and Sucrose in *Synechocystis*

**Figure 3:**
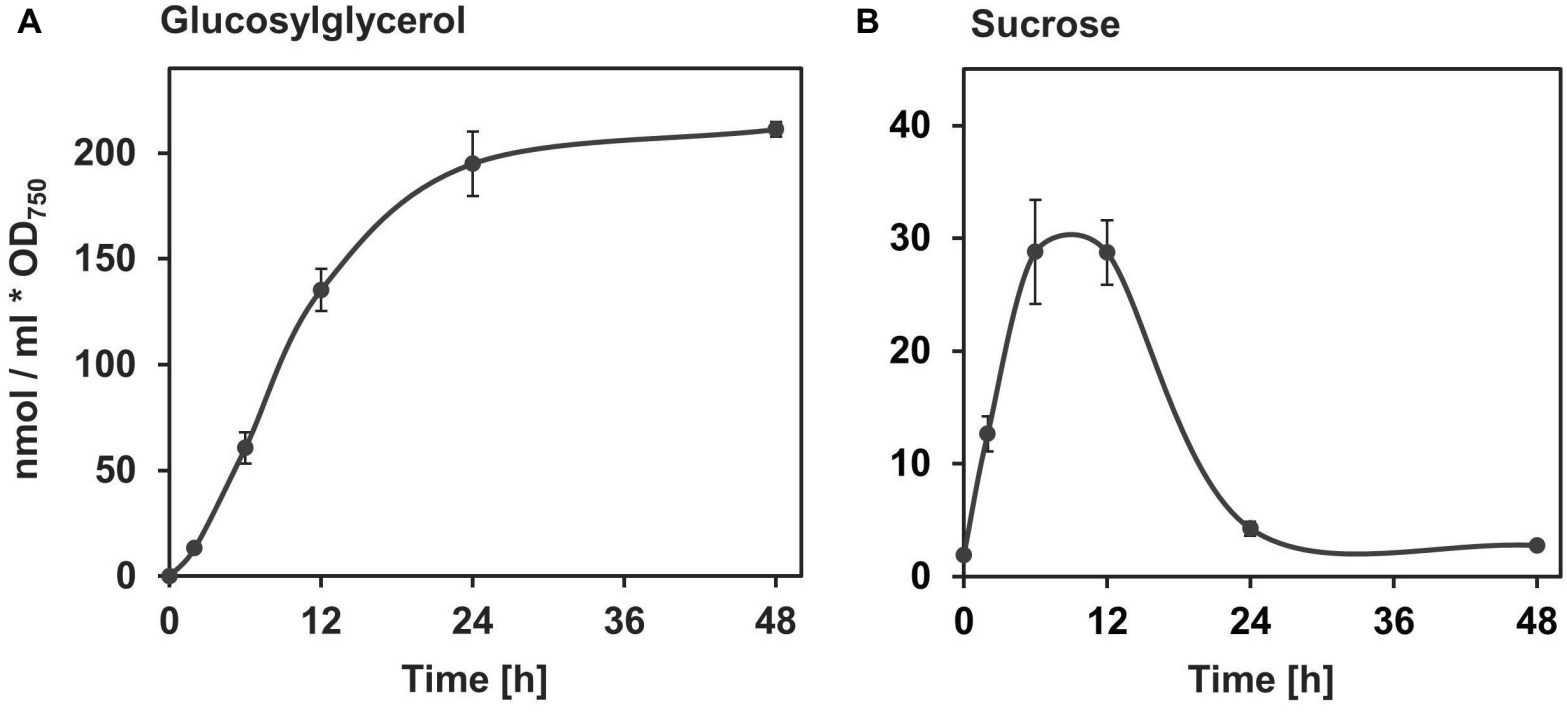
Salt-induced accumulation of the compatible solutes GG **(A)** and sucrose **(B)** in *Synechocystis* sp. PCC 6803. *Synechocystis* cells pre-cultivated in NaCl-free media were exposed to 3.5% NaCl (~ 600 mM, equivalent to full seawater conditions) at time point 0 and the compatible solutes were quantified *via* gas chromatography (Kirsch et al., 2017, 2018). The milliliter in the unit at the ordinate refers to culture volume.

In the model strain *Synechocystis*, the accumulation of GG and sucrose is induced immediately after a salt shock. In the case of GG, it reaches a final concentration after about 24 h, which then remains stable in salt-acclimated cells (Figure 3A) and is adjusted to the external salt level (Marin et al., 2002). Sucrose also accumulates rapidly in the cell within few hours (Figure 3B). However, this is a transient accumulation with a maximum of about 6–12 h after the salt shock (Desplats et al., 2005; Kirsch et al., 2018). As a result, the sucrose content drops again almost to the initial level within 24 h, likely due to the replacement by GG. In cyanobacteria of low salt tolerance, in which sucrose is the primary compatible solute (e.g., *Synechococcus elongatus* PCC 7942), this accumulation occurs to a final concentration and remains stable at that level thereafter (Blumwald et al., 1983).

### Activation of Glucosylglycerol-Synthesis After Salt Shock – Gene Expression and Biochemical Regulation

**Figure 4:**
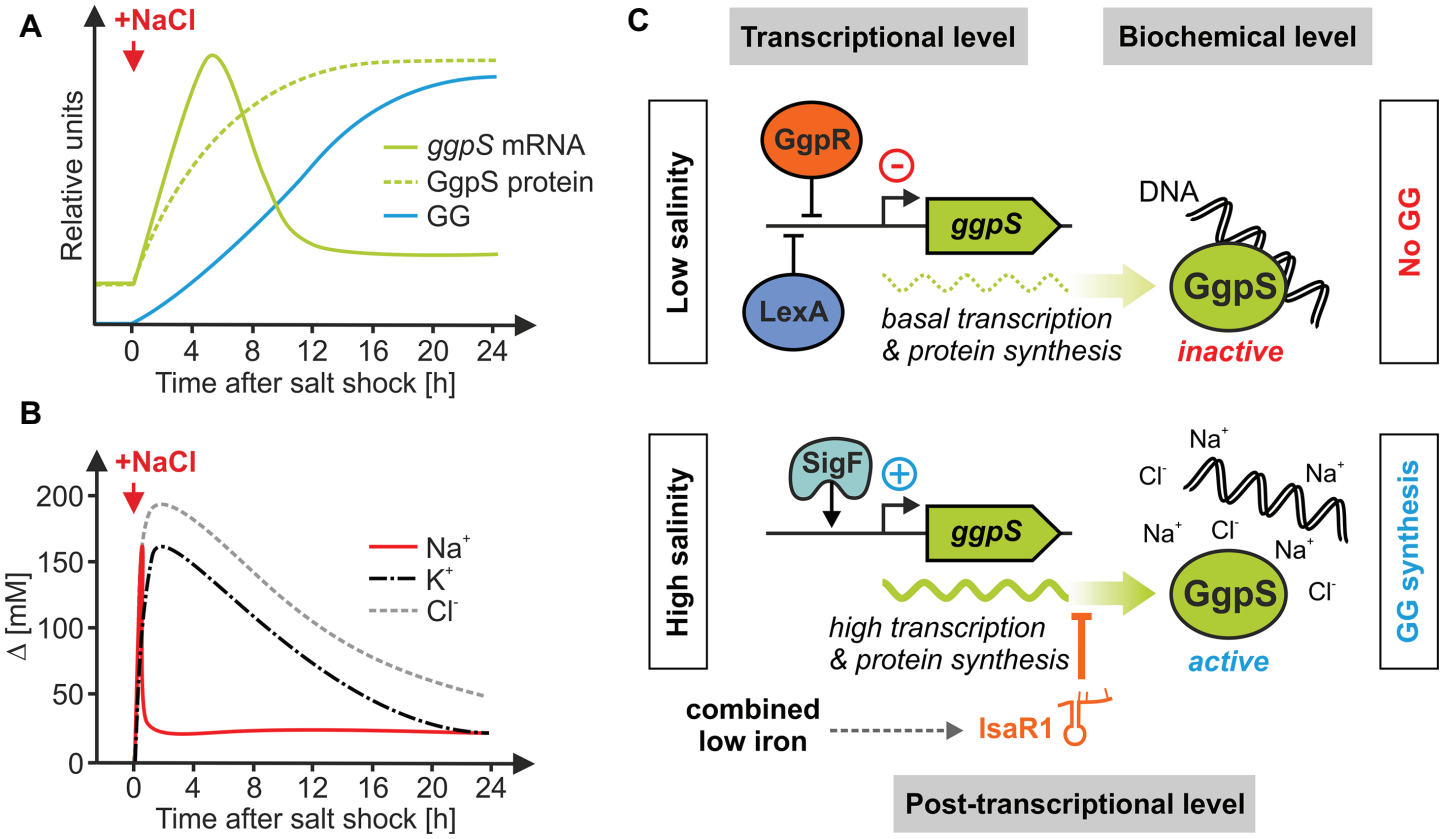
Regulation of salt-induced GG-synthesis *via* the key enzyme GgpS involves different regulatory layers in *Synechocystis*. **(A,B)** Kinetics of *ggpS* mRNA, GgpS protein, GG accumulation, and changes of salt ion concentrations after sudden salt shocks in *Synechocystis* [data have been obtained from Marin et al., 2002 **(A)** and Reed et al., 1985 **(B)**]. **(C)** Three proteins act on the transcriptional level, GgpR (Klähn et al., 2010b), LexA (Kizawa et al., 2016), and SigF (Huckauf et al., 2000; Marin et al., 2002). The sRNA IsaR1 interacts with *ggpS* mRNA at the postranscriptional level (Rübsam et al., 2018). The rapid activation of GG synthesis and its adjustment to the final salt content seem to be mainly regulated at the biochemical level *via* the cellular amount of inorganic ions (Novak et al., 2011).

Increases in salt content require a rapid and coordinated cell response. In many heterotrophic bacteria, the synthesis of compatible solutes is mainly activated at the transcriptional level, e.g., the salt-induced synthesis of proline is initiated by the transcriptional activation of the *proHJ* operon in *Bacillus subtilis* (Brill et al., 2011). This operon codes for the enzymes responsive for osmoadaptive proline synthesis, which is independent from proteins involved in the anabolic proline synthesis (Hoffmann and Bremer, 2016). In the case of GG, *Synechocystis* activates the pathway at both the biochemical and transcriptional level. Cells cultivated under NaCl-free conditions already contain a basal amount of inactive GgpS protein, which is activated very rapidly by a sudden increase in salinity. As it turned out, this biochemical activation takes place directly through NaCl or other salts (Hagemann and Erdmann, 1994). Since recombinant GgpS protein purified from *E. coli* has NaCl-independent activity, it was concluded that an inhibitor bound to GgpS is removed by NaCl *in vivo* (Hagemann et al., 2001). In fact, at low salinities, GgpS can be bound to nucleic acids due to electrostatic binding to the negatively charged phosphate groups, which results in an inactive enzymatical state (Novak et al., 2011). After salt shock, inorganic ions are flowing into the cell and can disrupt this electrostatic bond, which results in GgpS release and its activation. Thus, the initial biochemical activation of GG synthesis is mediated by the ions entering a cell upon salt addition, as has been shown for salt-shocked cyanobacteria (Figure 4; Reed et al., 1985; Hagemann et al., 1994). Interestingly, the salt-dependent activity of the *Synechocystis* GgpS was also observed when the gene was expressed in the heterologous host *Corynebacterium glutamicum* (Roenneke et al., 2018). In *Corynebacterium* as in *Synechocystis*, GgpS was active when cells were exposed to salt stress, however, in contrast to *Synechocystis* GG continuously leaked from the engineered *Corynebacterium* cells. The increases in salinity also induce *ggpS* gene expression in *Synechocystis* (Marin et al., 2004), thereby adjusting the amount of enzyme in addition to its activity regulation by changes in the salt content. Nevertheless, some strain-specific aspects should be noted here. For instance, in the marine strain *Synechococcus* sp. PCC 7002, the biochemical regulation of GgpS seems to play a minor role whereas the transcriptional induction of *ggpS* in salt-stressed cells is dominating (Engelbrecht et al., 1999).

Remarkably, the *ggpS* mRNA shows a salt-dependent accumulation that roughly follows the kinetics of the intracellular salt ion concentrations in response to salt shocks (Figures 4A,B). The induction of *ggpS* expression involves several regulatory components in *Synechocystis* (Figure 4C). First, on a superior level, the adjustment of *ggpS* transcription involves the use of alternative sigma factors. In *Synechocystis* for instance, the absence of the SigB and SigD factors lead to a decreased salt tolerance, which, however, is probably due to a generally reduced stress tolerance (Pollari et al., 2008) as found before by the knock out of the stress-regulatory sigma factors in *B. subtilis* (Hecker et al., 2007). A more specific role in salt acclimation appears to play SigF, because a *sigF* mutant strain shows markedly reduced *ggpS* expression accompanied by an impaired acclimation capacity to salt stress (Huckauf et al., 2000; Marin et al., 2002). Second, the genes of GG metabolism in *Synechocystis* have been reported to be under negative control of the transcription factor LexA, because the *lexA* mutant strain showed increased *ggpS* transcript levels already under low salt conditions (Kizawa et al., 2016). However, given the large number of LexA-regulated genes, this is likely more a global rather than a salt-specific regulation that interferes with GG synthesis in addition to various other cellular processes. Third, likely more GG-specific, regulatory functions are mediated at the transcriptional level by a small protein that is encoded by a gene located directly upstream of *ggpS*. It was shown that a deletion of this gene resulted in de-regulated *ggpS* expression. Hence, the respective protein appears to repress the expression of *ggpS* under low-salt conditions and was named *ggpS* repressor (GgpR) (Klähn et al., 2010b). It should be noted that clear homologs of GgpR are missing in the databases. However, a similar gene organization, namely a short open reading frame upstream of *ggpS,* was also found in a few other GG-accumulating cyanobacterial strains suggesting that such an organization rather than a conserved, DNA-binding repressor protein could be responsible for the salt-dependent transcriptional induction.

Moreover, the *ggpS* gene of *Synechocystis* was also shown to be target of post-transcriptional regulation by a small regulatory RNA (Figure 4C). The iron-stress activated RNA 1 (IsaR1) was previously identified as key regulator for adjusting the photosynthetic apparatus of various cyanobacteria to iron deficiency (Georg et al., 2017). This regulation is mainly conducted by complementary base-pairing between IsaR1 and its target mRNAs, which in turn interferes with protein biosynthesis, i.e., under iron-depletion when IsaR1 levels are strongly increased. Interestingly, the *ggpS* mRNA is also targeted by IsaR1. Thereby, IsaR1 clearly diminishes the *de novo* synthesis of GgpS protein as well as GG (Rübsam et al., 2018). However, the biological meaning of post-transcriptional *ggpS* regulation by a small RNA that is predominantly expressed under iron deficiency is not yet understood. Most likely, this is of importance when cells are exposed to parallel fluctuations in both salinity (triggering *ggpS* expression) and iron availability (triggering IsaR1 expression). In this regard, it should be noted that in natural environments, both situations frequently occur in parallel, e.g., in estuary brackish water habitats, where an increase in salinity is accompanied by a rapid removal of iron from river water (Boyle et al., 1977). Obviously, IsaR1 coordinates the response of *Synechocystis* to iron starvation with osmotic acclimation and hence is involved in the integration of responses to different environmental perturbations.

Altogether, GG accumulation in salt-shocked *Synechocystis* cells is regulated at different levels; however, the interplay and communication between these systems is largely unknown. The rapid start of GG accumulation immediately after salt shock seems to be mainly achieved *via* the direct activation of the basically synthesized GgpS protein. To ensure an efficient GG synthesis in salt-acclimated cells, an enhanced GgpS amount is necessary, which is apparently ensured by the release of the repressor GgpR and positive action of SigF. The GG synthesis must be imbedded into the overall carbon utilization network, which is probably regulated *via* LexA, since this transcriptional activator also affects the expression of many other genes involved in primary carbon metabolism (Kizawa et al., 2016). Finally, salt stress is often accompanied by multiple other stressors and the responses need to be integrated. This might be exemplified by IsaR1 that is likely part of a coordinated acclimation to simultaneous iron deficiency and salt stress frequently occurring in natural habitats.

### Activation of Sucrose-Synthesis After Salt Shock – Gene Expression and Biochemical Regulation

**Figure 5:**
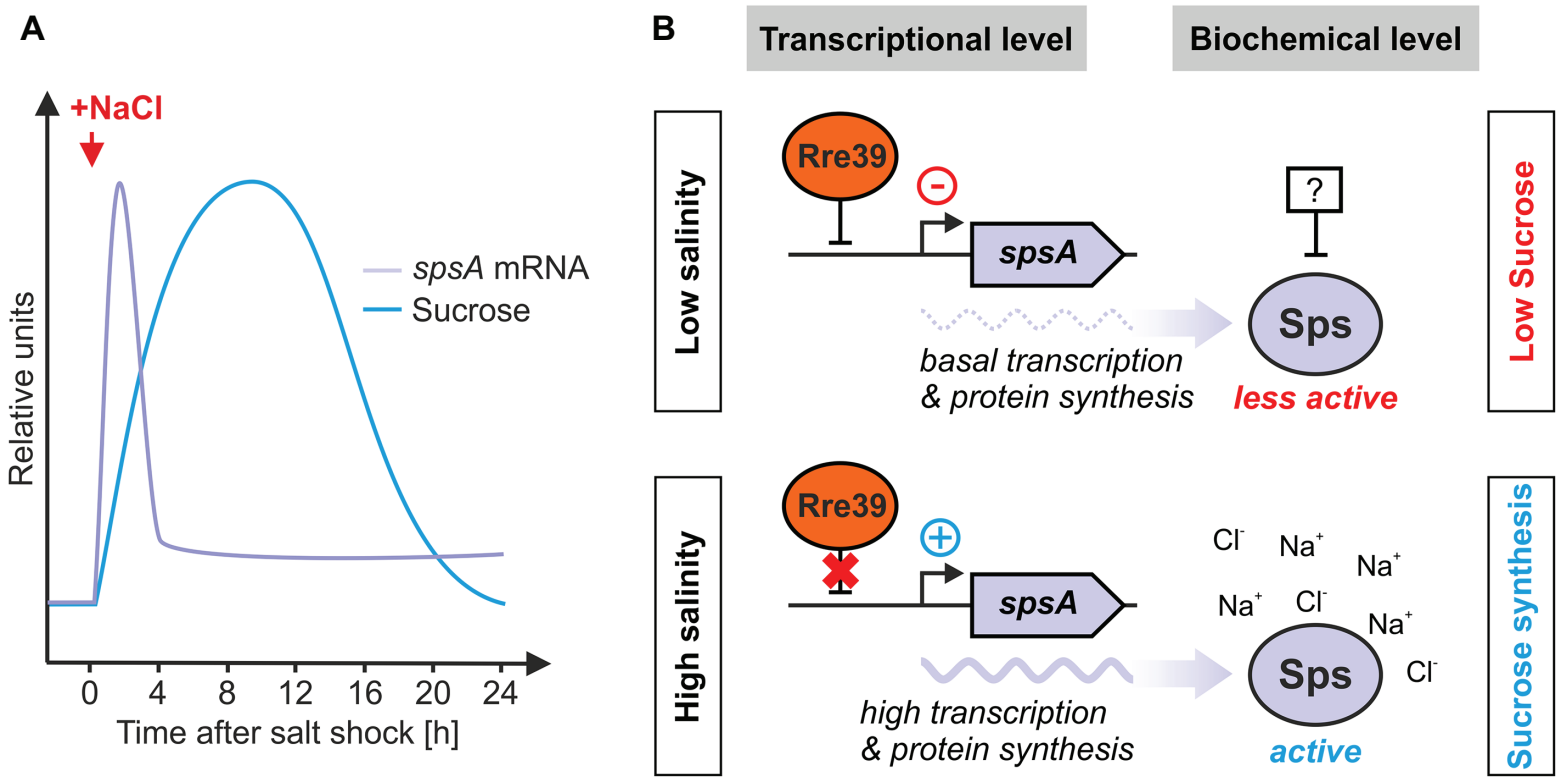
Regulation of salt-induced sucrose accumulation in *Synechocystis*. **(A)** The relative *spsA* mRNA and sucrose accumulation kinetics in response to a salt shock have been approximated from Marin et al. (2004) and Kirsch et al. (2018). **(B)** Regulation of expression and activity of sucrose-phosphate synthase by the response regulator Rre39 and the intracellular ion levels.

Compared to the regulatory mechanisms involved in the synthesis of GG, less is known for sucrose. However, in view of the very rapid accumulation of this sugar in salt-stressed cells of *Synechocystis*, a rapid activation of enzymes is to be expected here as well (Figure 5). Similar to GgpS in GG synthesis, Sps appears to be the rate-limiting enzyme in sucrose synthesis, as modifications of Sps expression have the greatest effect on the rate of sucrose accumulation (Du et al., 2013). Likewise, as with GgpS, stimulation of Sps activity by NaCl has been shown in *S. elongatus* PCC 6301 (Hagemann and Marin, 1999). However, this is not true for all Sps proteins, such *in vitro* stimulation could not be observed with the enzyme from *Nostoc* (*Anabaena*) sp. PCC 7120. These results suggest that within cyanobacteria, different regulatory aspects exist instead of a conserved mechanism for biochemical activation of sucrose synthesis. To which extent the biochemical activation of Sps is based on a protein-DNA interaction, similar to GgpS, is still unclear.

At the transcriptional level, the induction of *spsA* gene expression occurs within minutes after a salt shock (Marin et al., 2004; Desplats et al., 2005). However, this induction is significantly lower than for *ggpS* (induction factor of ~ 85 for *ggpS* versus a factor of ~ 3 for *spsA*, Marin et al., 2004). The factors involved in the transcriptional regulation of Sps are still largely unknown. Recently, the influence of response regulator 39 (Rre39; Slr1588) on Sps expression has been identified (Song et al., 2017). In mutant Δ*slr1588*, increased expression of *spsA* was observed, indicating a possible negative regulation by Rre39. Sucrose synthesis in salt-stressed cells of *Nostoc* (*Anabaena*) sp. PCC 7120 is regulated by the Rre named OrrA (Ehira et al., 2014). Rre’s are usually components of bacterial two-component signal transduction systems, which consist of a sensory histidine kinase (Hik) and a Rre and are also important sensor systems for various types of stress in cyanobacteria (Los et al., 2010). In *E. coli*, such two-component systems are also involved in the sensing of salt stress. The Hik/Rre pairs KdpD/KdpE (salt-dependent regulation of K^+^ uptake, Jung and Altendorf, 2002) and EnvZ/OmpR (salt-dependent regulation of permeability of the outer membrane, Mizuno and Mizushima, 1990) play a role here. *Synechocystis* Rre39, however, is an “orphan” response regulator, i.e., so far no associated Hik could be identified. Overall, five Hik/Rre systems have been reported that play a role in signal transduction under salt stress in *Synechocystis* (Marin et al., 2003; Shoumskaya et al., 2005). However, among the genes regulated, there are none that play a role in the synthesis of GG or sucrose. Hence, the signal transduction cascades triggering the transcriptional resonse of *ggpS* and *spsA* remain enigmatic.

## Response to Decreasing Salinities Involves the Degradation of Compatible Solutes

Unlike the accumulation of compatible solutes upon increasing salt levels, little is known about the cellular response of cyanobacteria to a decreasing salt concentration. Despite the opposite direction, salinity reduction may be just as challenging for a microorganism as it is an increase in salinity. In particular, due to the highly accumulated compatible solutes, the osmotic potential of the cytosol becomes lower compared to the extracellular space when salinity decreases. Accordingly, this leads to a flow of water into the cell that can represent a serious problem. For example, a hypoosmotic shock equivalent to 0.3 M has been shown to increase the turgor pressure from 4 (~400 kPa) to 11 atm (~1.1 MPa) in *E. coli* (Booth and Louis, 1999). This poses a dramatic burden on the cytoplasmic membrane and the cell wall. To prevent the cell from bursting, the particular organism must rapidly establish the osmotic balance between the cell’s interior and exterior. Two scenarios are conceivable for intracellularly enriched compatible solutes–either these are enzymatically degraded and metabolized or the intracellular substance concentration is adjusted by release from the cell. The latter represents a widely used strategy for adapting to rapidly and strongly decreasing salinity and is mediated by mechanosensitive channels (Levina et al., 1999). These channels are opened following a hypoosmotic shock, when the water influx induces a mechanical tension on the cell membrane exceeding a certain threshold. The enzymatic degradation of compatible solutes represents a slower response to less strong hypoosmotic conditions. Nevertheless, this strategy also has advantages, e.g., the large amounts of organic carbon (and sometimes nitrogen) incorporated into compatible solutes are retained for cellular metabolism. It is therefore not surprising that microorganisms have evolved precisely regulated ways of recycling compatible solutes internally.

### The Release of Compatible Solutes

When salt-acclimated cells of *Synechocystis* are exposed to a sudden hypoosmotic shock, i.e., when the salt concentration of the medium is lowered by at least 250 mM, large amounts of GG are immediately released into the medium (Fulda et al., 1990). This rapid reaction is likely relying on the opening of mechanosensitive channels (Msc) through which dissolved substances escape from the cell as shown for *E. coli* (Booth and Louis, 1999). The genome of *Synechocystis* contains eight candidate genes for homologs of MscS and one gene for MscL (Pivetti et al., 2003). So far, however, only the MscL (encoded by gene *slr0875*) has been demonstrated to function in adapting to hypoosmotic shocks (Nanatani et al., 2013). The mechanosensitive channels show a low specificity because, in addition to compatible solutes, a whole series of other dissolved substances such as inorganic ions and amino acids also escape into the medium (Fulda et al., 1990). Presumably, this release of solutes after a hypoosmotic shock is a rapid emergency reaction that prevents the cell from bursting due to osmotic water influx.

### Intracellular Degradation of Glucosylglycerol

In natural habitats such rapid changes in salinity are rather rare, but it could appear in mixing zones at sea shores and dry soils during heavy rain. Since the release of compatible solutes also results in a significant loss of previously fixed carbon, the existence of degradation pathways for the intracellular metabolisation of these substances can be assumed. In fact, compatible solutes in many bacteria also serve as storage substances for carbon and nitrogen (Welsh, 2000). This was demonstrated, for example, for the substances glycine betaine (Wargo, 2013), proline (Moses et al., 2012), and ectoine (Schulz et al., 2017). In cyanobacteria, specific evidence emerged from experiments, in which salt-adapted *Synechocystis* cultures were supplemented with the alternative compatible solute trehalose (Mikkat et al., 1997). It was found that the externally available trehalose was taken up and accumulated intracellularly. At the same time, the GG content in the cells decreased without GG being detected in the medium. These results suggested that GG was degraded intracellularly and replaced by the ingested trehalose. Another indication of a GG-degrading pathway was found in *Microcoleus chthonoplastes* (Moezelaar et al., 1996). Cultures that were grown in darkness and under anaerobic conditions performed fermentation processes in which GG was degraded in addition to glycogen. The glucose residue was fermented and the glycerol residue was released into the medium. Moreover, ^13^C-labeling experiments in *Synechococcus* sp. PCC 7002 revealed a strong exchange of labeled carbon between the pools of glycogen and GG (Baran et al., 2017). This indicates a continuous conversion of GG by synthesis and degradation at least in this strain.

**Figure 6:**
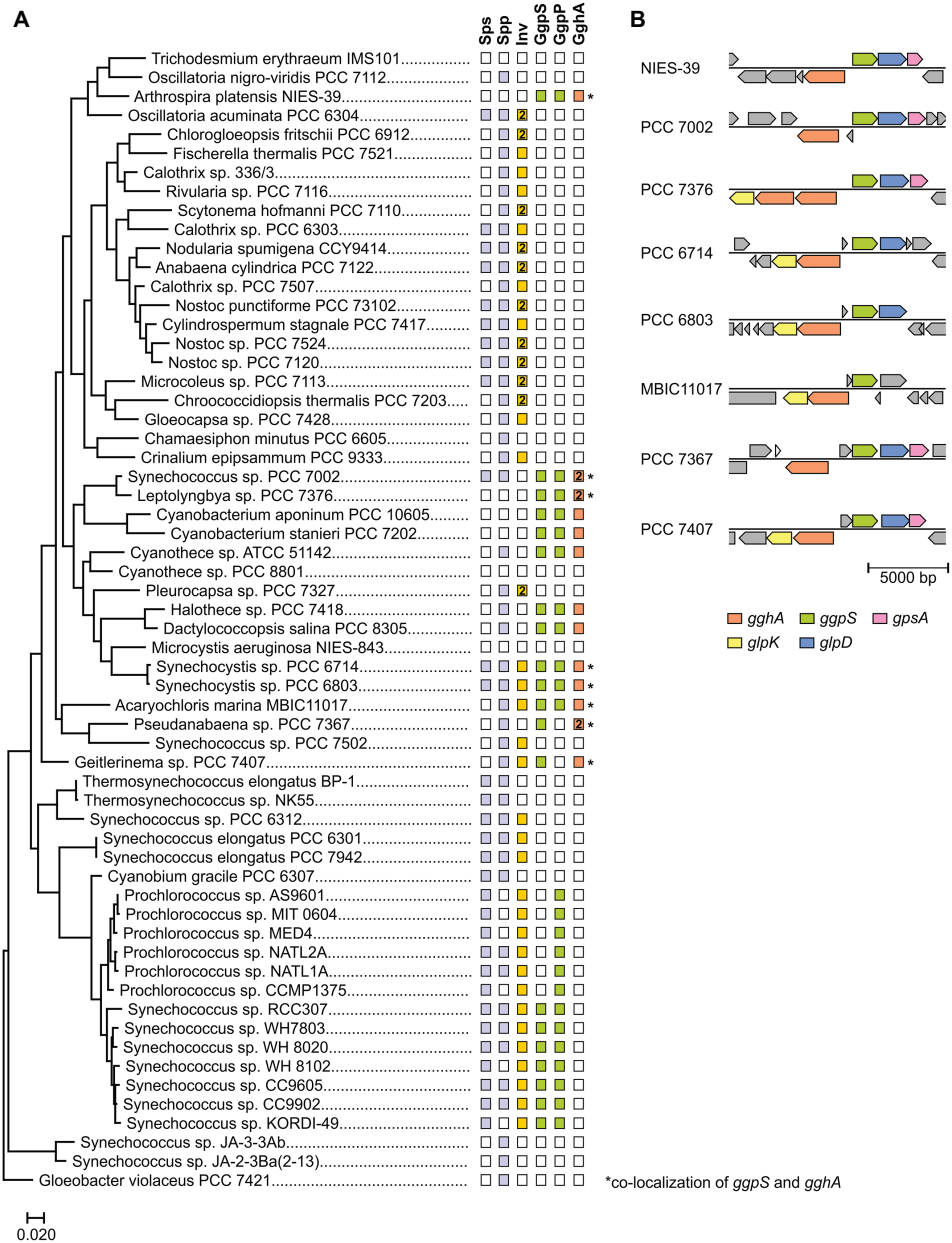
Distribution of GG-synthesis (*ggpS*) and GG-degradation (*gghA*) genes among cyanobacteria. **(A)** Phylogenetic tree of selected cyanobacteria based on 16S rDNA sequences. The tree was generated with the MEGA X (Kumar et al., 2018) software package and the Maximum Likelihood method. The presence of genes (illustrated by filled rectangle) was investigated using the BLASTP algorithm. As reference the amino acid sequences of functionally verified GgpS, GgpP, GghA, etc. from *Synechocystis* sp. PCC 6803 were used. **(B)** In various cyanobacteria *ggpS* and *gghA* are co-localized (*glpD*, glycerolphosphate dehydrogenase; *ggpS*, GG-phosphate synthase; *gghA*, GG hydrolase A; *glpK*, glycerol kinase; *gpsA*, glycerol 3-phosphate dehydrogenase).

The above mentioned studies suggested that GG-synthesizing cyanobacteria also have a degradation pathway for GG and hence, its intracellular recycling. Recently, the responsible enzyme glucosylglycerol hydrolase A (GghA) was identified in *Synechocystis.* This enzyme hydrolyzes GG into glycerol and glucose (Savakis et al., 2016; Kirsch et al., 2017). It could be shown that GghA is responsible for the depletion of intracellular GG in response to decreasing salinities and thus represents a significant element of the cyanobacterial salt acclimation process (Kirsch et al., 2017). Interestingly, in the genome of *Synechocystis*, the respective gene *gghA* (*slr1670*) is located upstream of the *ggpS* gene, however on the complementary DNA strand (Figure 6). The promoters of both genes overlap in the region encoding the small regulatory protein GgpR (Klähn et al., 2010b). Transcriptome analyses verified a salt-dependent expression of *gghA* (Marin et al., 2004). The *gghA* gene is likely co-transcribed with the genes *glpK* (*slr1672*) encoding glycerol kinase and *spoU* (*slr1673*) that encodes a putative tRNA/rRNA methyltransferase. GlpK plays a role in the formation of glycerol 3-phosphate and thus enables reassimilation of the released glycerol back into primary metabolism. The co-localization of *gghA* with *ggpS* and *glpK* appears to be conserved among GG-synthesizing strains (Figure 6).

With the hydrolytic cleavage of GG into the products glucose and glycerol, GghA differs from the previously identified GG-splitting enzyme GG-phosphorylase (GGP) from *Bacillus selenitireducens*, which catalyzes the reversible cleavage of GG into glucose-1-phosphate and glycerol (Nihira et al., 2014). Interestingly, the activity of GghA becomes increasingly inhibited by adding inorganic salts to *in vitro* enzyme assays (Kirsch et al., 2017). Moreover, a Δ*gghA* mutant of *Synechocystis* showed a clear phenotype, as this strain lost its ability to adjust the GG content to decreasing salt concentrations. This was demonstrated by two experiments in which salt-adapted cultures of the wild type and the Δ*gghA* mutant were exposed to either a gradual reduction in salinity or an abrupt hypoosmotic shock. Continuous dilution of the salinity over several hours did not result in the release of GG into the medium, but the wild type simultaneously adjusted the intracellular GG concentration to the altered salinity. This was not the case in the Δ*gghA* mutant, in which the amount of GG remained unchanged (Kirsch et al., 2017).

Moreover, GghA also plays an important role in the acclimation to sudden hypoosmotic shock treatments. As mentioned above, after such a treatment, both the wild type and the Δ*gghA* mutant strain release GG into the culture medium. However, GG quickly disappears from the medium and cells of wild type, whereas it remains in supernatants of the mutant Δ*gghA* (Kirsch et al., 2017). It has been shown that an ABC-type transporter named Ggt is involved in the uptake of GG but also trehalose and sucrose when externally provided to the cells. Mutation of the transporter resulted in the continuous release of GG into the medium, which gave raise to the hypothesis that the main function of the Ggt system is related to the re-uptake of compatible solutes leaked out the cells to avoid losses of organic carbon (Hagemann et al., 1997b; Mikkat and Hagemann, 2000). Correspondingly, the released GG after hypoosmotic shocks is likely re-imported into cells of the wild type by the Ggt system, where it is hydrolyzed by GghA and the glucose is then used to provide carbon and energy supporting the quick acclimation to the new growth conditions. In contrast to wild type, the Δ*gghA* mutant was unable to use the released GG and showed a decreased ability to restart growth after hypoosmotic treaments. These experiments illustrated that the GG degradation is crucial for the acclimation to fluctuating salinities.

The importance of GghA is underlined by the fact that several GG synthesizing cyanobacteria even harbor two copies of the *gghA* gene. However, a *gghA* gene is absent from marine picocyanobacterial strains of the genus *Synechococcus* all of which harbor *ggpS* and *ggpP* genes (Figure 6). These genes are functional and lead to intracellular GG accumulation, which has been verified, e.g., for *Synechococcus* sp. WH7803 and WH8102 (Klähn et al., 2010a). The lack of a GG-degrading pathway in these strains, however, could be explained by a rather constant saline environment in the open oceans and the needlessness of osmotic adjustments in response to decreasing salinities. Nevertheless, an alternative, so far unknown GG-degradation pathway cannot be excluded.

In addition to the described defect in GG degradation, another interesting phenotypical change was observed for the strain lacking GghA. The cultures of the Δ*gghA* mutant became very viscous when grown under NaCl-free conditions, suggesting the release of extracellular polysaccharides (EPS) from the cells (Kirsch et al., 2017). It is therefore of interest to investigate a possible role of GG or its intermediate GGP in the synthesis of these substances. Research on the complex functions of EPS in biofilm formation and adaptation to different types of stress have become increasingly interesting in recent years (Pereira et al., 2009).

### Intracellular Degradation of Sucrose

Within cyanobacteria, three different degradation pathways for sucrose have been identified so far (Kolman et al., 2015). The responsible enzymes are sucrose synthase, amylosucrase and invertase (Inv). Sucrose synthase can catalyze both synthesis and cleavage of sucrose, but appears to be active primarily in the direction of the cleavage reaction to (A/U)DP-glucose and fructose *in vivo* (Porchia et al., 1999; Curatti et al., 2002). This enzyme is mainly found in heterocyst-forming cyanobacteria and plays an essential role for N_2_-fixation (Kolman et al., 2015). Amylosucrase has recently been described in *Synechococcus* sp. PCC 7002 (Perez-Cenci and Salerno, 2014), where it splits sucrose into free fructose and glucose, the latter is then linked *via* glycosidic bond to oligosaccharides or glycogen. In most cases, however, the degradation of sucrose among cyanobacteria occurs by the enzyme Inv, which catalyzes the irreversible cleavage of sucrose into the monosaccharides glucose and fructose. Phylogenetic studies suggested that invertases were part of the original sucrose metabolism and were transferred from cyanobacteria into plants (Vargas and Salerno, 2010). The invertases form a large and diverse group of sucrose-cleaving enzymes. A biochemical classification can be made on the basis of the pH optimum for enzyme activity, distinguishing invertases with a pH optimum in the acid range from pH 4.5 to 5 (acid invertases, Ac-Inv) and invertases having an optimum in the neutral to slightly alkaline range from pH 6.5 to 8 (alkaline/neutral invertases; A/N-Inv). Phylogenetically, Ac-Inv and A/N-Inv are unrelated. The Ac-Inv are β-fructofuranosidases (EC 3.2.1.26) that are not only specific for sucrose but can also cleave other β-fructose-containing oligosaccharides such as raffinose and stachyose (Sturm, 1999). They occur mainly in heterotrophic bacteria, yeasts, and plants; e.g., in plants, they are extracellularly bound to the cell wall or localized in the vacuole. Ac-Inv are of great importance for the food industry and hence, have been extensively studied (Nadeem et al., 2015). The occurrence of A/N-Inv is, however, limited to cyanobacteria and plants (Vargas et al., 2003). These enzymes differ from the Ac-Inv not only in terms of their pH optimum, but also catalytically. Thus, they do not belong to the family of β-fructofuranosidases because they are specific for sucrose and do not cleave other β-fructose-containing substrates (Vargas and Salerno, 2010). Recently, the crystal structure of the alkaline invertase from *Anabaena* (InvA) has provided clues for the catalytic mechanism of these enzymes. The A/N Inv of cyanobacteria and plants specifically cleave the α, β-1,2-glycosidic linkage of sucrose and represent a distinct family of glucosidases (Xie et al., 2016).

The role of sucrose degradation by the enzyme Inv in salt acclimation of *Synechocystis* has recently been investigated (Kirsch et al., 2018). Similar to most unicellular cyanobacteria (Kolman et al., 2015), also the genome of *Synechocystis* harbors one gene that is annotated as A/N-Inv (CyanoBase–http://genome.annotation.jp/cyanobase). To gain evidence, the respective gene *sll0626* was expressed in *E. coli.* The recombinant protein was able to cleave sucrose *in vitro*, with an optimum at pH 7. In the slightly alkaline region at pH 8, the activity of Sll0626 was reduced by 23%. These data show that Sll0626 is a neutral invertase, named *Synechocystis* invertase (SyInv) (Kirsch et al., 2018). The measured Km for sucrose of 14.7 mM is in good agreement with the invertases from *Anabaena* (36 mM for A-Inv and 15 mM for N-Inv, Vargas et al., 2003) and plants (10 mM for A/N-Inv, Sturm, 1999). Interestingly, the activity of SyInv becomes increasingly inhibited by adding inorganic salts to *in vitro* enzyme assays. The activity was almost halved at 75 mM NaCl and reached only about 15% of the initial level at 145 mM NaCl (Kirsch et al., 2018). These inhibitory ion concentrations are markedly exceeded in salt-stressed cells of *Synechocystis* (Hagemann et al., 1994).

The mutation of SyInv provided strong evidence that this protein is responsible for *in vivo* sucrose degradation in *Synechocystis* (Kirsch et al., 2018). Unlike the wild type, the Δ*inv* mutant already showed significant amounts of sucrose when grown under NaCl-free conditions, which indicates active sucrose turnover and explains the low steady state content of sucrose in wild-type cells. The addition of 3.5% NaCl induced the synthesis of sucrose in the wild type as well as in the mutant. However, sucrose enrichment was not transient in the Δ*inv* mutant. In contrast to the sucrose accumulation kinetics in wild type (see Figure 3), the Δ*inv* mutant showed constantly enhanced sucrose content also after long-term salt acclimation (Kirsch et al., 2018). Hence, the quick decrease of sucrose accumulation in salt-shocked wild-type cells is mainly based on the action of sucrose degradation *via* SyInv.

### Biochemical Model of the Salt-Dependent Activation of Glucosylglycerol and Sucrose Accumulation in *Synechocystis*

**Figure 7:**
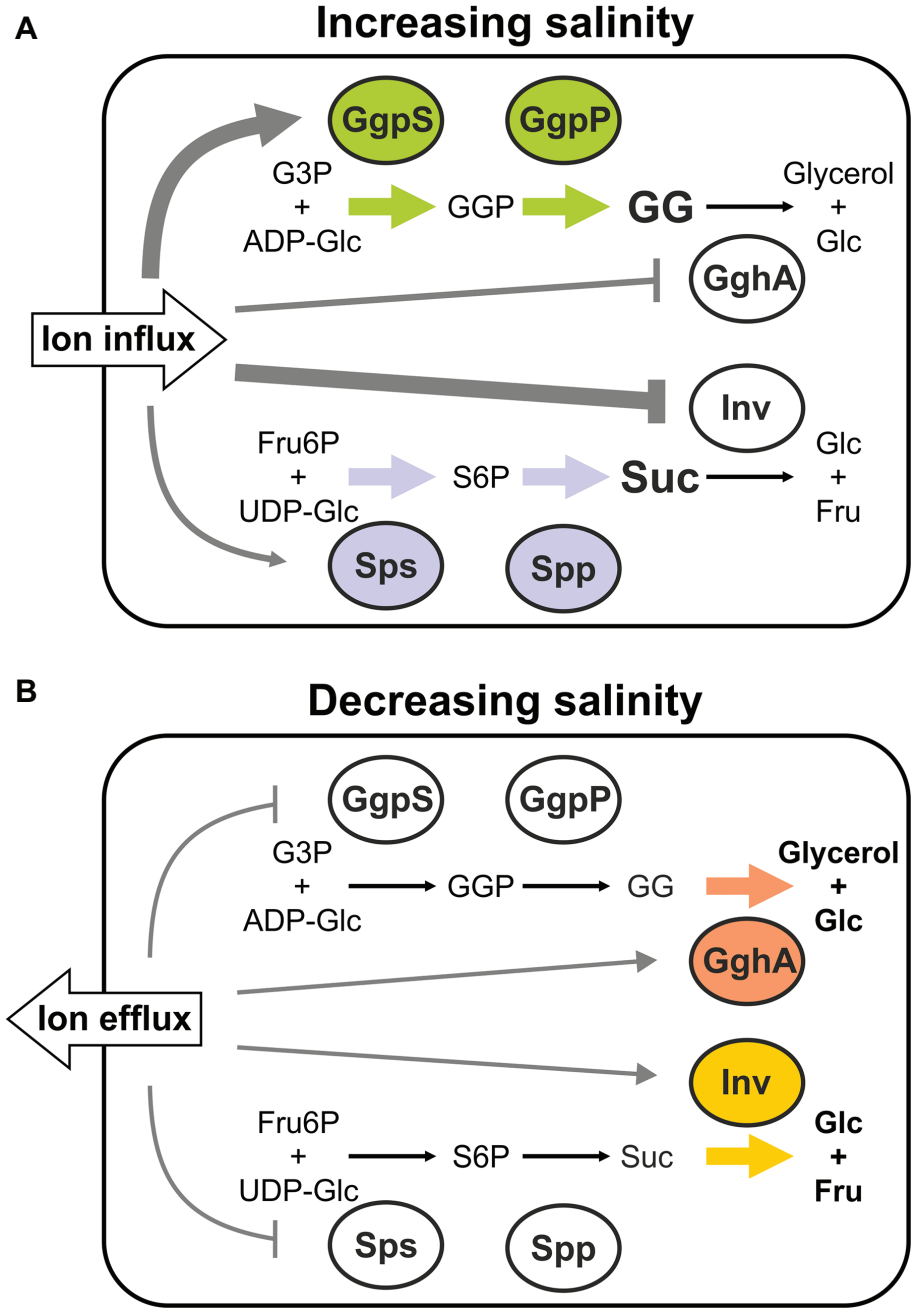
Biochemical regulation of GG and sucrose accumulation in salt-treated cells of *Synechocystis* sp. PCC 6803 *via* inorganic ions. The hypothetical model is based on the different response of corresponding synthesis and degradation enzymes toward changing inorganic ion contents. The increase **(A)** or decrease **(B)** of external salinity results in the influx or efflux of inorganic ions, which in turn modulate the activities of enzymes involved in GG and sucose biosynthesis and degradation, respectively. (GgpS, GG-phosphate synthase; GgpP, GG-phosphate phosphatase; GghA, GG hydrolase A; Sps, sucrose-phosphate synthase; Spp, sucrose-phosphate phosphatase; Inv, invertase; G3P, glycerol 3-phosphate; ADP-Glc, ADP-glucose; GG, glucosylglycerol; GGP, GG-phosphate; Glc, glucose; Fru, fructose; Fru6P, fructose 6-phosphate; UDP-Glc, UDP-glucose; S6P, sucrose 6-phosphate; Suc, sucrose).

The biochemical data obtained for compatible solute synthesizing as well as degrading enzymes suggest opposite controlling in cyanobacteria. On the one hand, increased inorganic ion amounts (represented by NaCl) stimulate the activity of enzymes for the synthesis of GG and sucrose *in vitro*. On the other hand, the activity of degrading enzymes is inhibited by higher concentrations of inorganic ions. Based on these findings we suggest a biochemical model for the control of compatible solute accumulation in *Synechocystis* (Figure 7). Exposure to enhanced salinities immediately activates the accumulation of sucrose and GG due to ion-mediated enzyme activation. It has been shown that Na^+^ ions move inside the cells within seconds, and are later exchanged by K^+^, while compatible solute biosynthesis starts only thereafter within minutes (Reed et al., 1985, see Figure 4B). While GgpS and probably also Sps activities are activated, the degradation enzymes GghA and SyInv are switched off at the same time. This inverse enzyme behavior may result in the observed rapid initial GG and sucrose accumulation (Figure 7).

At a later stage, the osmotic balance between the cell interior and the exterior is increasingly achieved by the accumulated compatible solutes, which permits to reduce the intracellular inorganic ions. According to our model, the decrease of the intracellular ion pool is responsible for the decreased sucrose accumulation after 12 h, because lower ion contents result in lowered Sps activity but particularly increased SyInv activity. The predominant effect of increased Inv activity is supported by the finding that the decrease of the sucrose pool is almost absent in mutant Δ*inv* (Kirsch et al., 2018). The synthesis of GG continues with maximum speed for longer times until the steady state level is reached. Probabably the decreased GgpS activity in the presence of lowered intracellular ion contents is compensated by the massive expression stimulation of *ggpS* after salt shocks (Marin et al., 2004). In this context, the sucrose content is preferably regulated by the ion-mediated regulation of its degradation by the invertase, whereas for the high and continuous GG accumulation the ion-mediated activation and synthesis of GgpS is of greater importance in salt-shocked cells of *Synechocystis* (Figure 7).

Despite the active export, long-term salt-acclimated cells still have a higher steady state content of inorganic ions. For example, the intracellular Na^+^ concentration in *Synechocystis* increases from 32 mM in cells cultivated under NaCl-free conditions to 70 mM or even 215 mM when grown in media supplemented with 2 or 4% (w/v) additional NaCl (Hagemann et al., 1994). It can therefore be assumed that GghA is only slightly active due to these inhibitory ion concentrations in salt-adapted cells, whereas a sufficient part of the large GgpS pool is released from the inhibitory binding of DNA to synthesize GG (Figure 7).

### Biotechnological Utilization of Salt-Induced Glucosylglycerol Accumulation

The interest in GG as a biotechnological product is based on its applicability in the pharmaceutical and cosmetic sector. It can serve as a stabilizer for the storage of enzymes and antibodies (Borges et al., 2002; Luley-Goedl et al., 2010). In skin care products, it is used as an additive due to its hydrating and revitalizing effect and marketed under the product name Glycoin^®^ by the bitop AG. Presently, GG is commercially synthesized by the enzyme sucrose phosphorylase (from *Leuconostoc mesenteroides*), which converts the cheap precursors sucrose and glycerol *via* a transglycosylation reaction into GG and fructose (Luley-Goedl et al., 2010). However, it is conceivable to establish a GG production directly from cyanobacterial cultures, e.g., as byproduct from biocatalysts used for biofuel production in saline media. For example, optimized production strains of *Synechocystis* can produce approximately 981 mg GGL^-1^ (Tan et al., 2015). To achieve this high productivity, the genes *ggtC* and *ggtD* (uptake of GG) as well as *ggpR* (repressor of GG synthesis) were mutated. It has been previously shown that high GG yields can be obtained from wild type by a cyclic cultivation, during which the cultures are alternately exposed to salt stress for GG synthesis and hypoosmotic shocks that release the previously enriched GG (Reed et al., 1986; Erdmann et al., 1992). Using such a strategy and the optimized producer strain, it was possible to effectively harvest the produced GG in up to four consecutive production cycles (24 days in total) (Tan et al., 2015). Continuous release of GG without hypoosmotic shocks would require an effective exporter. A candidate gene for such a protein is *ycaD* from *Stenotrophomonas rizophila* (Roder et al., 2005; Alavi et al., 2013). Unfortunately, an exact characterization of the corresponding protein is still pending, but the expression of *ycaD* in GG-producing cyanobacteria may potentially lead to continuous export and high GG titers (Pade and Hagemann, 2015). Recently, a GG-producing *Corynebacterium glutamicum* strain has been engineered using the GG synthesis genes from *Synechocystis*, which continuously released GG into the culture medium and produced under optimized conditions more than 10 mM GG (approximately, 2 g L^−1^) (Roenneke et al., 2018). It has been further reported for Synechocystis that mutation of the *slr1670* gene (later annotated as *gghA*) resulted in increased cellular GG accumulation (Savakis et al., 2016). In our own work, however, no significantly increased steady state concentration of GG could be confirmed in salt-adapted cultures of the Δ*gghA* mutant (Kirsch et al., 2017). Moreover, GG production has been also achieved with transgenic potato plants, which accumulated 10 μmol GGg^-1^ FM in leaves and up to 2.6 μmol GGg^-1^ FM in tubers (Sievers et al., 2013).

### Biotechnological Use of Sucrose

With respect to biotechnology, salt-induced sucrose production has also moved into focus, because it is an attractive nutritive sugar and feedstock (Hagemann and Hess, 2018). The potential of cyanobacterial sucrose production has been studied to date with the organisms *Synechococcus elongatus* PCC 7942 (Ducat et al., 2012), *Synechocystis* (Du et al., 2013; Kirsch et al., 2018), and *Synechococcus elongatus* UTEX 2973 (Song et al., 2016). The expression of sucrose permease (sucrose/proton symporter, CscB) from *E. coli* proved to be very effective for sucrose production in both *S. elongatus* strains, PCC 7942 and UTEX 2973. In these freshwater strains, sucrose is the only compatible solute, and continuous loss *via* CscB resulted in a dramatic increase in sucrose synthesis when cultured in saline media (Ducat et al., 2012). Approximately, 35 mg sucrose L^−1^ h^−1^ were produced by strain *Synechococcus* CscB during cultivation at 200 mM NaCl. The authors performed a scaling up calculation of potential annual cyanobacterial sucrose production rates, which showed that cyanobacterial sucrose yields could be similar to those obtained with sugar cane (Ducat et al., 2012).

In *Synechocystis*, the sucrose content could be increased by the mutation of *ggpS* blocking GG synthesis and thereby enabling increased carbon flux in sucrose (Du et al., 2013; Kirsch et al., 2018). In addition, it has been shown that overexpression of the Sps also contributes to increasing sucrose synthesis (Du et al., 2013). As discussed above, the Δ*inv* mutant of *Synechocystis* also has an increased sucrose content (Kirsch et al., 2018). It should be emphasized as a unique feature of this mutant that there are significant amounts of sucrose in the cells even under salt-free culture conditions. Based on this, the inactivation of the invertase was carried out in the strain WD25, which represented the most potent sucrose-producing *Synechocystis* strain (Du et al., 2013). Even with this genetic background, the *inv* mutation proved to be very effective, as in the resulting strain QL240, a 40% increase in sucrose production was observed compared to the parental WD25 (Kirsch et al., 2018). Thus, using the example of sucrose, the potential of genetic modifications to metabolically direct carbon into a desired product becomes apparent. However, it remains to be verified if sucrose from cyanobacterial production will be able to compete with traditionally obtained sucrose from sugar cane and sugar beet in future. Compared to the established method for obtaining sucrose from the plant material, a purification from cyanobacterial cultures appears to be labor-intensive and therefore cost-intensive, because sucrose must be separated from NaCl of the used saline growth medium. Hence, the future use of sucrose-producing cyanobacteria in co-cultivation with heterotrophic microorganisms seems more promising as has been also suggested by Ducat et al. (2012) and Löwe et al. (2017). Here, the sugar obtained from photosynthesis can serve as carbon and energy source for the synthesis of high value substances. The advantage of those systems is the elimination of downstream processing, i.e., a purification of sucrose from cells or the medium is not required. The work of Ducat et al. (2012) showed the positive effect of the sucrose-exporting *S. elongatus* PCC 7942 on the growth of yeast. Such a strain was also proven to be an effective co-cultivation partner for a polyhydroxyalkanoate-producing *Pseudomonas putida* strain (Löwe et al., 2017).

## Outlook

Our knowledge on cyanobacterial GG and sucrose biosynthesis has been strongly improved during recent years, particularly the aspect of compatible solute degradation has come into focus of current research. The biochemical characterization of GG and sucrose synthesizing as well as degrading enzymes allowed us to propose a simple biochemical model to describe the ion-mediated accumulation and consumption of these compatible solutes in the model strain *Synechocystis* under hyperosmotic and hypoosmotic salt conditions, respectively (see Figure 7). Contrary to older views, labeling studies even revealed that compatible solutes are not accumulated as a dead end of metabolism but rather show a quick turnover–at least observed for the compatible solute GG in *Synechococcus* sp. PCC 7002 (Baran et al., 2017). Other studies indicated a close connection between sucrose and glycogen pools in salt-stressed cells of *S. elongatus* (Qiao et al., 2018). Nevertheless, one important question remains unanswered: to which extent can the knowledge obtained from *Synechocystis* or other well-investigated models transferred toward different cyanobacterial strains on the one hand and even toward other compatible solutes on the other hand? For example, differences in the relative importance of biochemical or transcriptional regulation of GG synthesis seem to exist between euryhaline strains or stenohaline such as marine strains adapted to constantly high salinities. Moreover, there are hints that similar to GgpS other enzyme activities are also stimulated in salt-stressed cells (e.g., Sps in *Synechocystis*). However, it is completely uncertain whether or not the model of inhibitory electrostatic interaction of GgpS with inhibitory DNA, which can be influenced by changed intracellular ion concentration (Novak et al., 2011) may play a more general role. Hence, it would be interesting to study a larger spectrum of cyanobacterial strains regarding GG as well as sucrose synthesis, for example to identifiy enzymes depending or not depending on salt for their activity, which would permit to analyze the structural basis for the salt-mediated enzyme regulation and also provide compatible solute synthesis enzymes that could be active in the absence of high NaCl amounts improving downstream processing.

In addition to the direct regulatory effects of ions on enzyme activities, the regulatory circuits involved in the sensing of salt stress and the signal transduction triggering gene expression programs are still not well understood. It has been proposed that instead of specific two-component systems or sigma factors, the changed ion and metabolic signature, e.g., accumulated K^+^ glutamate, might influence the specificity of promoter recognition by RNA polymerase (Gralla and Vargas, 2005). However, recently, the second messenger nucleotide cyclic-diAMP has been identified as potential signal molecule involved in the sensing of salt stress signals and crucial for K^+^ homeostasis (e.g., Nelson et al., 2013).

The closure of these knowledge gaps is not only of academic interest. As mentioned above, the increasing utilization of cyanobacteria for biotechnological applications will depend on mass cultivation of suitable strains in saline media to save limited freshwater resources. For sure, strain-specific salt acclimation modes and their regulatory circuits need to be revealed to minimize negative effects of stress acclimation on product formation. Moreover, there might be a broader spectrum of compatible solues that can be used for different pharmaceutical or other commercial purposes as high value byproducts of cyanobacterial mass cultivation. Hence, the selection of new cyanobacterial chassis organisms and the introduction of new biosynthetic routes for valuable bioproducts should always include an integrated study of salt acclimation and product synthesis under fluctuating salinities.

## Author Contributions

All authors listed have made a substantial, direct and intellectual contribution to the work, and approved it for publication.

### Conflict of Interest Statement

The authors declare that the research was conducted in the absence of any commercial or financial relationships that could be construed as a potential conflict of interest.
